# DNAdigest and Repositive: Connecting the World of Genomic Data

**DOI:** 10.1371/journal.pbio.1002418

**Published:** 2016-03-24

**Authors:** Nadezda V. Kovalevskaya, Charlotte Whicher, Timothy D. Richardson, Craig Smith, Jana Grajciarova, Xocas Cardama, José Moreira, Adrian Alexa, Amanda A. McMurray, Fiona G. G. Nielsen

**Affiliations:** 1 DNAdigest, Future Business Centre, Cambridge, United Kingdom; 2 Repositive Ltd, Future Business Centre, Cambridge, United Kingdom

## Abstract

There is no unified place where genomics researchers can search through all available raw genomic data in a way similar to OMIM for genes or Uniprot for proteins. With the recent increase in the amount of genomic data that is being produced and the ever-growing promises of precision medicine, this is becoming more and more of a problem. DNAdigest is a charity working to promote efficient sharing of human genomic data to improve the outcome of genomic research and diagnostics for the benefit of patients. Repositive, a social enterprise spin-out of DNAdigest, is building an online platform that indexes genomic data stored in repositories and thus enables researchers to search for and access a range of human genomic data sources through a single, easy-to-use interface, free of charge.

## Abstract 2

This Community Page article introduces the charity DNAdigest, which aims to promote best practices for ethical and efficient genomic data sharing, and the free online community platform Repositive, for easy search and access to genomic datasets.

## Genomic Data Sharing: Hurdles and Needs

### The Root of the Problem

Irrespective of whether biomedical research is funded publicly or privately, there is increasing pressure to provide evidence that the maximum benefit is obtained from generated data. This pressure is increasing not only from funding agencies but also from the patient community and individual data donors, who expect their data to be used in an efficient and ethical way.

While it is acknowledged that more effective data reuse and reanalysing between and across research studies would be able to reduce false positive results, increase chances of novel discoveries and reliability of research outputs [1–5], it currently largely falls to the individual custodian of the data to decide how to share it with the research community (if at all). Even when funding bodies explicitly require data sharing, the current hurdles to data discoverability and access often keep “shared data” unavailable in a practical sense.

In light of our recent blog post by Prof. Barbara Prainsack from King’s College London, available at http://tinyurl.com/dnadigest-sharing, we would like to define more specifically what we mean by data sharing. When we talk about facilitating data sharing in this article or elsewhere, we mean improving (1) discoverability, (2) access, and (3) reuse of genomic data.

Recent years have seen a concerted effort by providers of public funds for research to require that the results of that research be publicly available [6]. This effort has been largely focused on preventing publicly funded research papers from being locked behind paywalls. However, efforts have also been made to extend the application of these principles to require the availability of research data.

The logical conclusion of these moves towards reform of publicly funded data reuse is the requirement that researchers make data as widely available as can be achieved within the consent given by the data donor.

### Data Sharing: A Common Hurdle in Scientific Research

There are several subjective and objective reasons why researchers do not make their data available [7]: “My data contains personal and/or sensitive information; my data is too complicated; people may misinterpret my data; my data is not very interesting; commercial funders do not want to share it; we might want to use it in a(nother) paper; people will contact me to ask about stuff; Data Protection/National security issues; my data is too big; people will see that my data is bad; I want to patent my discovery; it is not a priority and I am busy; I do not know how to share data; I am not sure I own the data; someone may steal or plagiarise it; my funder does not require it.”

Why do research scientists need to share data? Firstly, it ensures transparency and reproducibility, which is a source of ongoing concern [1,2]. Furthermore, more routine data sharing would increase the availability of complementary and/or reference datasets, saving time and resources [8] and opening up the possibilities for new discoveries.

### Genomic Data Sharing: A Special Case

While the benefits of data sharing are becoming more widely accepted [3,4], human genomic data (i.e., information about the composition of our DNA and RNA) is often exempt from major funders’ data sharing requirements that all experimental data must be placed in publicly accessible repositories. This is because of concerns that making human genomic data public exposes potentially sensitive, personal information to the world [5].

**Fig 1:**
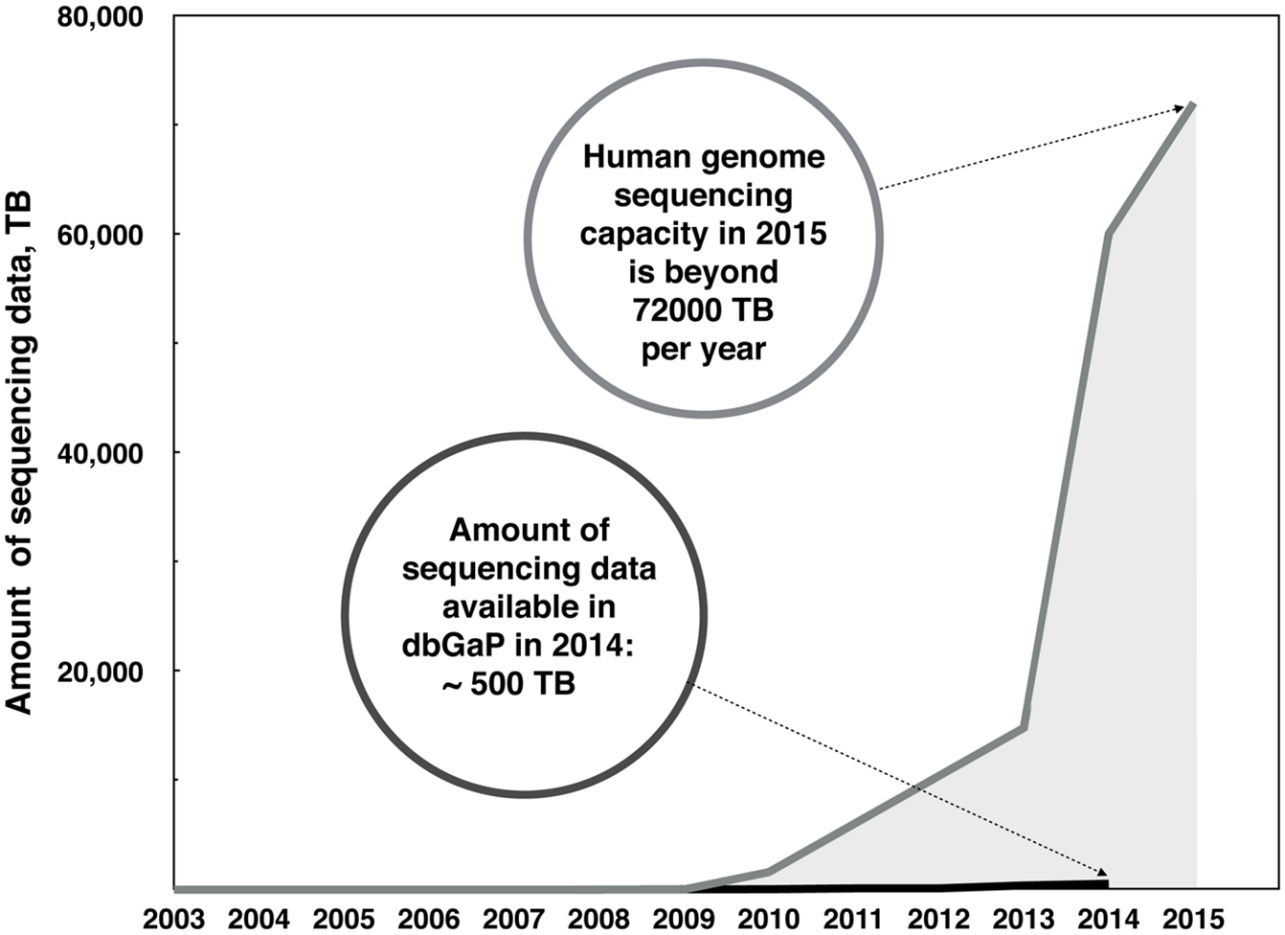
Estimated minimum annual human genome sequencing capacity based on sales of Illumina HiSeq X annual throughput capacity (at least 16 systems sold worldwide) and the amount of data available up to 2014 via dbGaP—one of the largest repositories for clinical human genomic data [12]. Taking into account that a whole genome sequence is ~200 GB in size, this corresponds to ~360,000 and ~3,000 human genomes, respectively [9,10,13].

It is estimated that, in 2015, the world human genome sequencing capacity will exceed 80 petabytes of sequence a year [9–11]. However, as of 2014, the largest public repository for human genomic data (the NIH database of genotypes and phenotypes: dbGaP [12]) holds only about 0.5 petabytes of clinical genomic data (Fig 1). This number is calculated based on the amount of sequencing data in the largest restricted-access human genomic data repository, dbGaP [12]. As of 20 March 2014, dbGaP contained 534,691,127,128,640 bytes of information under restricted access, which we assume to be clinically relevant information and/or whole human genomes. Assuming that the size of a whole genome sequencing experiment for a human genome has a storage footprint of ~200 GB, the amount of clinical data in dbGaP is the data size equivalent of ~2,673 whole human genome datasets at 30x sequencing coverage.

This gap between the availability of genomic information and the production of it can be at least partially attributed to the absence of tangible benefits for the individuals who make data available and, at the same time, to the existence of sanctions for improper handling of personal information. However, when data donors give consent for their data to be used for research, they set their expectations that the data will actually be used for this purpose. To not utilise their data in the best possible way within the consent given goes against the data donor’s interests and expectations.

Ironically, human genomic data is probably the most important data to share, since it lies at the heart of efforts to combat major health issues such as cancer, genetic diseases, and genetic predispositions for complex diseases like heart disease and diabetes. In particular, the promise of personalised medicine (in which treatment is tailored to the individual) is unlikely to be realised without widespread access to large amounts of genomic data.

In a previous study [8], we researched the barriers for efficient data sharing and identified the common steps in workflows of genomics researchers in different settings (academic, clinical, and commercial researchers). Each of those workflows included searching for external data. Currently, there are approximately 20 public repositories containing different types of genomic data (cf. Tables 1 and 2). Most of the public repositories are “open access,” while others require an application to a data access committee (e.g., European Genome-Phenome Archive [EGA] database of Genotypes and Phenotypes [dbGaP]), which slows down the process dramatically. Many researchers agree that data access applications are complex and time-consuming and the process suffers from most repositories being too complicated and inconsistent in the way they present information. Our interviews revealed that, on average, every research scientist is familiar with 4.5 repositories and uses them regularly. Most researchers have 1–2 repositories that they use, and if they cannot find the necessary data in their preferred repositories they usually give up instead of searching elsewhere. The reasons for this behaviour are (1) the lack of centralised information about different resources, (2) poor structure and annotation of data in the repositories, and (3) the amount of time required to apply for restricted access datasets.

**Table 1:** A list of repositories where researchers can download or upload genomic data.

Repository	Data Types	Description	URL
**dbGaP** ^a^	Raw sequence data and phenotypic data	Database of Genotypes and Phenotypes, developed to archive and distribute the results of studies that have investigated the interaction of genotype and phenotype.	http://www.ncbi.nlm.nih.gov/gap
**dbVar**	Variant data	Database of genomic structural variation—it contains insertions, deletions, duplications, inversions, multinucleotide substitutions, mobile element insertions, translocations, and complex chromosomal rearrangements.	http://www.ncbi.nlm.nih.gov/dbvar
**dbSNP**	Variant data	Database of single nucleotide polymorphisms (SNPs) and multiple small-scale variations that include insertions and deletions, microsatellites, and non-polymorphic variants.	http://www.ncbi.nlm.nih.gov/snp
**GEO**	Raw sequencing data	Public functional genomics data repository supporting Minimum Information About a Microarray Experiment (MIAME)-compliant data submissions. Tools are provided to help users query and download experiments and curated gene expression profiles.	http://www.ncbi.nlm.nih.gov/geo/
**Sequence Read Archive (SRA)**	Raw sequencing data	Stores raw sequencing data and alignment information from high-throughput sequencing platforms.	http://www.ncbi.nlm.nih.gov/sra
**ClinVar**	Variant data	Aggregates information about genomic variation and its relationship to human health.	http://www.ncbi.nlm.nih.gov/clinvar/
**The European Genome-phenome Archive (EGA)** ^a^	Raw sequence data and phenotypic data	Allows you to explore datasets from genomic studies, provided by a range of data providers.	https://www.ebi.ac.uk/ega/
**The European Nucleotide Archive (ENA)**	Raw sequencing data	A comprehensive record of the world's nucleotide sequencing information, covering raw sequencing data, sequence assembly information and functional annotation.	http://www.ebi.ac.uk/ena
**The European Variation Archive (EVA)**	Variant data	An open-access database of all types of genetic variation data from all species.	http://www.ebi.ac.uk/eva/
**ArrayExpress**	Raw sequencing data	Archive of Functional Genomics Data stores data from high-throughput functional genomics experiments, and provides these data for reuse to the research community.	https://www.ebi.ac.uk/arrayexpress/
**DNA data bank of Japan (DDBJ)** ^a^	Raw sequencing data	Collects nucleotide sequence data as a member of the International Nucleotide Sequence Database Collaboration (INSDC) and provides freely available nucleotide sequence data and supercomputer system, to support research activities in life science.	https://www.ddbj.nig.ac.jp
**Japanese Genotype-phenotype Archive (JGA)** ^a^	Raw sequencing data	A service for permanent archiving and sharing of all types of individual-level genetic and de-identified phenotypic data resulting from biomedical research projects. The JGA contains exclusive data collected from individuals whose consent agreements authorize data release only for specific research use or to bona fide researchers.	https://trace.ddbj.nig.ac.jp/jga/index_e.html
**Catalogue of somatic mutation in cancer (COSMIC)** ^a^	Variant data	Stores and displays somatic mutation information and related details and contains information relating to human cancers. There are two types of data in COSMIC: expert manual curation data and systematic screen data.	http://cancer.sanger.ac.uk/cosmic
**DECIPHER** ^a^	Variant data and phenotypic data	Database contains data from >17,800 patients who have given consent for broad data-sharing. Used by the clinical community to share and compare phenotypic and genotypic data.	https://decipher.sanger.ac.uk
**Figshare**	Raw sequencing data	A repository where users can make all of their research outputs available in a citable, shareable, and discoverable manner.	http://figshare.com
**Dryad**	Raw sequencing data	A curated resource that makes the data underlying scientific publications discoverable, freely reusable, and citable. Dryad provides a general-purpose home for a wide diversity of datatypes.	http://datadryad.org
**LOVD** ^a^	Variant data	A free, flexible, Web-based, open source database developed and designed to collect and display variants in the DNA sequence.	http://www.lovd.nl/3.0/home
**GigaDB**	Raw sequencing data	Associated with the journal GigaScience, contains discoverable, trackable, and citable datasets that are available for public download and use.	http://gigadb.org
**The Autism Genetic Resource Exchange (AGRE)** ^a^	Variant data and phenotypic data	A repository of biomaterials and phenotypic and genotypic data to aid research on autism spectrum disorders.	http://agre.autismspeaks.org
**Genomes unzipped (GNZ)**	Raw sequencing data	A collaborative project aiming to provide genetic testing customers with the knowledge and tools they need to make the most of their own genetic data. As part of the project, members are taking commercial genetic tests and making the raw data publicly available for others to download, analyse, and reuse.	http://genomesunzipped.org
**OpenSNP**	Raw sequencing data	Allows individuals to publish their genetic test results, find others with similar genetic variations, learn more about their results, get the latest primary literature on their variations, and help scientists find new associations.	https://opensnp.org

^a^ Restricted access repositories.

**Table 2:** A list of downloadable genomic data collections.

Repository	Data Types	Description	URL
**Exome Aggregation Consortium (ExAC)**	Raw sequencing data	A coalition of investigators seeking to aggregate exome sequencing data from a wide variety of large-scale sequencing projects and to make summary data available for the wider scientific community.	http://exac.broadinstitute.org
**The Cancer Genome Atlas (TCGA)** ^a^	Raw sequencing and phenotypic data	Comprehensive genomic characterisation and analysis of various cancers.	http://cancergenome.nih.gov
**International Cancer Genome Consortium (ICGC)** ^a^	Variant data	Comprehensive description of genomic, transcriptomic, and epigenomic changes in 50 different tumour types and/or subtypes that are of clinical and societal importance across the globe.	https://icgc.org
**1000 Genomes**	Raw sequencing data	The first project to sequence the genomes of a large number of people, to provide a comprehensive resource on human genetic variation.	http://www.1000genomes.org
**ENCODE**	Raw sequencing data	Aiming to build a comprehensive parts list of functional elements in the human genome.	https://genome.ucsc.edu/ENCODE/
**Exome Variant Server**	Raw sequencing data	National Heart, Lung and Blood Institute (NHLBI) Exome Sequencing Project (ESP) aims to discover novel genes and mechanisms contributing to heart, lung, and blood disorders by applying next-generation sequencing of the protein coding regions of the human genome and to share these datasets and findings with the scientific community.	http://evs.gs.washington.edu/EVS/
**Personal Genome Project**	Raw sequencing data	A group of research studies creating freely available scientific resources that bring together genomic, environmental, and human trait data donated by volunteers.	http://www.personalgenomes.org
**The Genome of the Netherlands**	Raw sequencing data	The Dutch biobank collaboration BBMRI-NL has initiated the extensive Rainbow Project “Genome of the Netherlands” (GoNL) to build a global genetic profile of large numbers of Dutch.	http://www.nlgenome.nl
**Simons Genome Diversity Project Dataset** ^a^	Raw sequencing data	A dataset of diverse, high-quality human genome sequences.	https://www.simonsfoundation.org/life-sciences/simons-genome-diversity-project-dataset/
**University of California Santa Cruz (UCSC) genome browser**	Raw sequencing data	This site contains the reference sequence and working draft assemblies for a large collection of genomes.	https://genome.ucsc.edu/

^a^ Restricted access repositories.

## Our Approach

### Acknowledging the Problems

In 2013, the initiative DNAdigest was founded and shortly thereafter registered as a charity in the United Kingdom by Fiona Nielsen, a bioinformatician who previously worked in research and development (R&D) at Illumina. DNAdigest was established with an aim to explore the problematic topic of genomic data sharing, engage the research community in discussions about the problems and potential solutions, and to build tools to incentivise and increase data access and reuse in genomics research.

The regular activities of DNAdigest include: (1) running hack days and workshops at which best practices and tools for more efficient and ethical data sharing are identified and discussed (see, for example, http://tinyurl.com/dnadigest); (2) promoting existing data sharing initiatives, tools, and organisations by featuring them in the DNAdigest blog, newsletter, and social media; (3) researching the current challenges for data sharing and disseminating the research results [8]. DNAdigest particularly aims to engage with graduate students, in the hope that discussions and education about best practices in data sharing will contribute to bringing up a new generation of well-informed, collaborative researchers who will take initiative to share data responsibly.

Through its activities, DNAdigest identified the major problems that genomics researchers are facing: all researchers complain about (1) the lack of available data, (2) cumbersome user-unfriendly interfaces, (3) difficulties with accessing restricted datasets, and (4) poorly and sometimes incorrectly annotated data. Furthermore, most of the interviewees wanted to have access to raw genomic data in order to analyse it independently in order to validate their research hypotheses.

### Providing Potential Solutions

In order to be independent of temporary funds and develop a long-term sustainable and scalable impact for the research community, the decision was made to spin out a social enterprise that would build software tools for efficient use of genomic data, aligned with the mission of DNAdigest. In 2014, DNAdigest spun out Repositive, a limited company that builds software and tools to facilitate the workflows of data access and data sharing across the research communities in academia and industry.

It was fundamental for Fiona to keep DNAdigest and Repositive independent, the former as a registered charity, the latter as a self-sustained business with its mission aligned to the social mission of the charity: “We registered DNAdigest as a charity to cement the mission of our project, and we are reaching out to collaborate with all relevant stakeholders and support all companies and initiatives that enable efficient and ethical data sharing. The charitable model is great for mission alignment, but for raising funding for our software development activities, we chose to spin out Repositive as a separate social venture.” The synergy and relationship between Repositive and DNAdigest is defined in Repositive’s Articles of Association, ensuring that Repositive upholds its mission statement “to facilitate efficient and ethical data sharing for genomics research” and that its employees are enabled to support DNAdigest activities through their work.

### Repositive: The Data Discovery Platform

**Fig 2:**
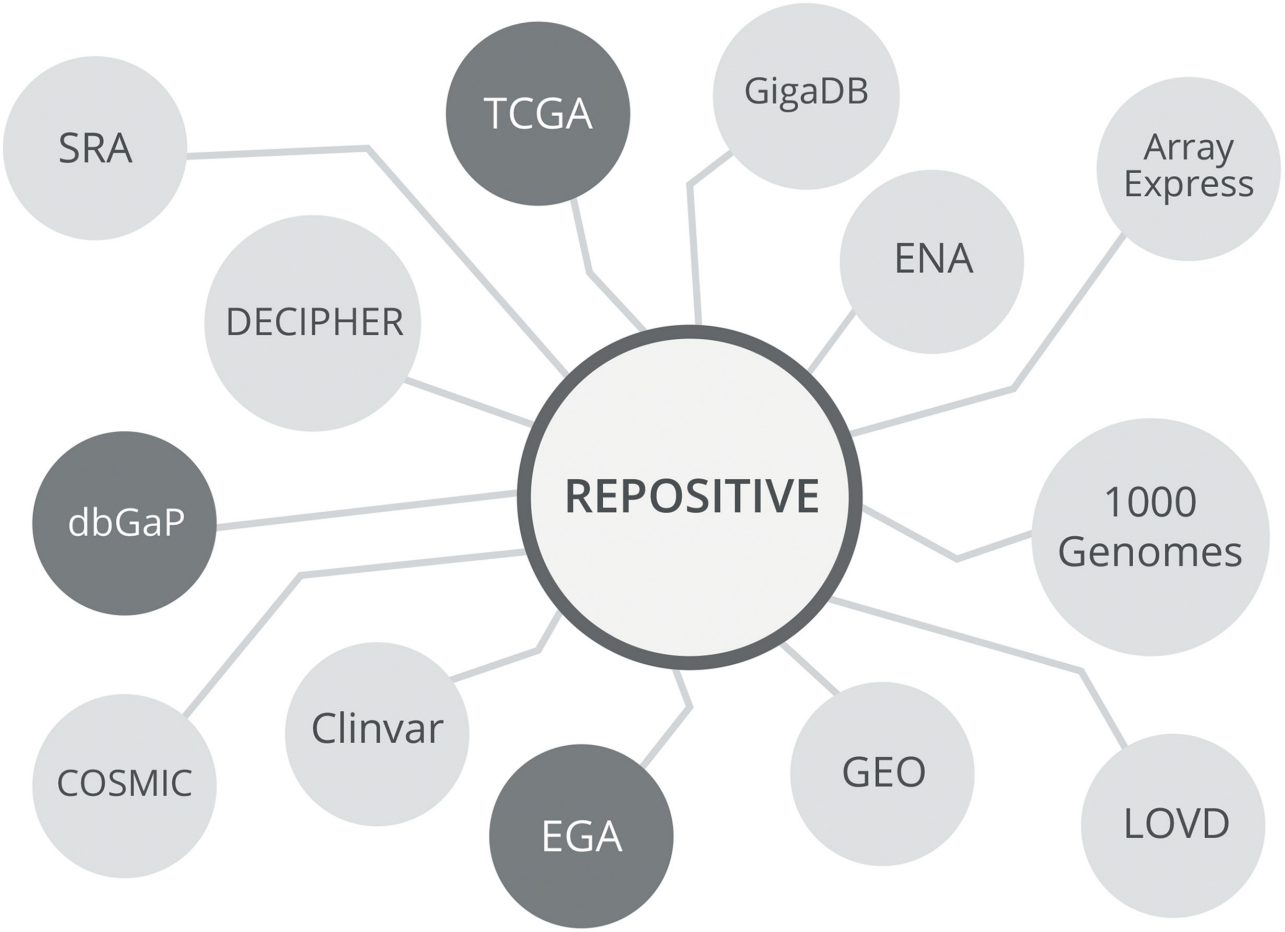
Repositive is an online platform indexing public human genomic data repositories. It enables registered users to find, access, and share genomic data that is consented for research use.

To address the most pressing problem for public genomic data: that of data discoverability, Repositive has built an online platform (repositive.io) providing a single-point entry to search public genomic data repositories (Fig 2).

The Repositive platform enables users to search through all its indexed data sources in a single click via an easy-to-use interface free of charge. One can think of the platform as an online portal and community for finding, accessing, and sharing of public genomic data: a one-stop shop to find the location of the most relevant data for researchers’ needs. Importantly, the Repositive platform holds descriptions and metadata but does not store the data itself: the user can click through to the source for data access.

### Repositive: The Community Platform

Given the facts that there is no single standard that all repositories follow, that different ontologies are used by different repositories, and that metadata annotations are provided to different levels of detail, locating and indexing data from existing repositories turns out to be a challenging task.

To address the problem of varying quality and type of metadata associated with data in public repositories, the Repositive platform allows users to comment on the content and quality of datasets and add descriptions to the listed metadata. For example, suppose, a researcher applied for restricted access datasets, waited for several months, downloaded the data and found out that the filenames had been transposed. By leaving a comment about the dataset, the researcher can save someone else a lot of effort trying to work out what is wrong. At the same time, the researcher sharing this additional information about a dataset does not break any rule imposed on him/her by the repository and this does not impact privacy issues.

**Fig 3:**
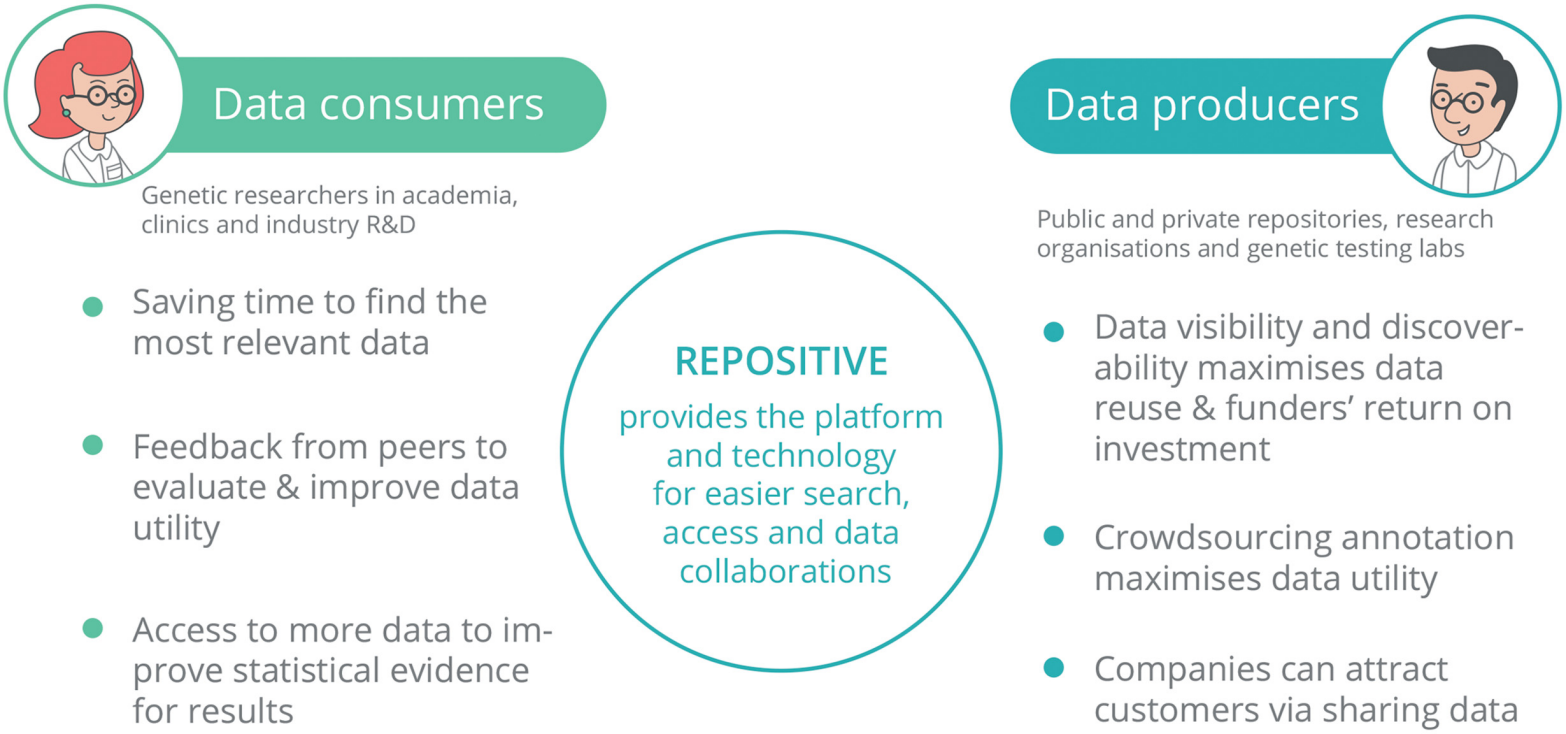
The Repositive platform provides benefits for both sides of the data exchange.

If a research scientist has data that he/she would like to share but cannot for any reason, he/she can announce the existence of the data on the Repositive platform. In this case, other scientists that have similar or complementary data can contact the author to start a collaboration or to discuss the conditions under which they can exchange their data, for example. Similarly, a user can post a request for data and another user, who has the data stored but not used, can respond and find an application for their otherwise useless data (Fig 3).

## Discussion

Since DNAdigest was founded in 2013, we have organised between four and five public events a year to bring the challenges of research data sharing in genomics to the attention of the research community as well as the general public. Through our workshops, hack days, and symposia, we have brought to light numerous approaches and solution models which have been actively debated and tested by our attendees. Many existing tools and initiatives for data sharing have been featured in our events, online blog, newsletter, and social media, and we continue to actively reach out to projects and initiatives to let them share their insights and best practices with the research community. We continue to find that efficient and ethical data sharing remains a challenging and multi-faceted issue, but it is encouraging to see that both tools and policies to support best practices are on the rise.

Through the mission-led spin-out of DNAdigest, the company Repositive, we are working to address some of the challenges, especially in relation to simplifying data discoverability and data access mechanisms. Repositive offers products and services that facilitate data discovery and efficient data access across repositories and data collaborations for researchers in both academia and industry.

The online Repositive platform currently has two main goals: (1) to facilitate data discoverability and access to genomic research data in public repositories, (2) to facilitate data collaborations within the genomics research community. Of these two goals, data discoverability using openly available metadata is the necessary and required first step to enable more data access and data collaborations with minimal risk to data privacy. The latter is important because even simple access mechanisms to privacy-sensitive data may provide risks to privacy if the access is not combined with governance mechanisms [13].

There are a number of online communities that feature networking and collaboration opportunities for scientists. These include Researchgate, Academia.edu, and LinkedIn. Each of these platforms allows researchers to interact with each other and to build their online profiles, which might help them find collaborations. There are also several existing projects that address data sharing problems by providing online open access repositories for storing and sharing research outputs. These include but are not limited to Figshare, Zenodo, and Dryad. All these platforms allow data storage, data sharing, and data annotation. The online communities and the data storage tools mentioned above all have a very broad coverage and are actively used by many researchers across very different fields of research.

However, there is currently no single point of entry for genomic datasets (like Uniprot for proteins or OMIM for genes). The Repositive platform incorporates a number of collaborative features and (meta)data annotation tools and is focused on improving data access and reuse specifically within human genomics research. Repositive is not a data repository, but rather a portal to search through various data locations, including the aforementioned repositories. At the same time, Repositive offers social tools similar to the community platforms mentioned above. Researchers can strengthen their profiles by providing data locations and annotations, thus building their online profile and increasing their chance of finding like-minded data collaborators.

Repositive is building a worldwide community of genomic researchers who are seeking data access solutions. We are building partnerships with both data providers and data consumers to overcome the hurdles that prevent data from being re-used for best possible impact within the given consent for data usage. This includes servicing both academic and industry organisations to set up platforms for data sharing, including the user interface, technology, and governance systems for setting up pre-competitive collaborations around genomic data.

We believe that by concentrating on one specific problem (in our case, the problem of finding and accessing human genomic research data) and supporting best practices for data annotation, accessibility, and reuse, the Repositive platform and services can contribute significantly to the field of genomic data sharing.

There are multiple other problems that need to be addressed to make data sharing the default rather than the exception. These include the standardisation of ethics committee approvals, normalising file formats, defining suitable ontologies, and metadata formats for describing data. Many of these issues are addressed by working groups within a number of international consortia, including the Research Data Alliance, BioSHaRE-EU, and the Global Alliance for Genomics and Health, of which both DNAdigest and Repositive are members.

## Conclusions

DNAdigest investigates the barriers for ethical and efficient data sharing for human genomic research and engages with all stakeholder groups, including researchers, librarians, data managers, software developers, policy makers, and the general public interested in genomics. We welcome new ideas for events and ways to reach out to the research community to embrace best practices for data sharing.

Repositive offers services and tools that reduce the barriers for data access and reuse for the research community in academia, industry, and clinics. To address the most pressing problem for public genomic data, that of data discoverability, Repositive has built an online platform (repositive.io) providing a single point of entry to public genomic data repositories. The Repositive platform is now in beta-testing and we welcome new users to come and try it out.
